# Novel Insights into Pontocerebellar Hypoplasia Type 3: Discovery of a New Disease-causing PCLO Variant and Development of a CRISPR-generated Cell Model: Novel Insights into Pontocerebellar Hypoplasia Type 3

**DOI:** 10.31661/gmj.v14i.3727

**Published:** 2025-08-10

**Authors:** Maryam Baneshi, Sedigheh Mohammadi, Hossein Jafari Khamirani, Jafar Fallahi, Mahintaj Dara, Fatemeh Sadat Tabei, Maryam Ranjbar, Seyed Mohammad Bagher Tabei

**Affiliations:** ^1^ Department of Medical Genetics, Shiraz University of Medical Sciences, Shiraz, Iran; ^2^ Department of Medical Biotechnology, School of Advanced Medical Sciences and Technologies, Shiraz University of Medical Sciences, Shiraz, Iran; ^3^ Student Research Committee, Shiraz University of Medical Sciences, Shiraz, Iran; ^4^ Department of Molecular Medicine, Shiraz University of Medical Sciences, Shiraz, Iran; ^5^ Stem Cells Technology Research Center, Shiraz University of Medical Sciences, Shiraz, Iran; ^6^ Maternal-fetal Medicine Research Center, Shiraz University of Medical Sciences, Shiraz, Iran

**Keywords:** PCLO, Piccolo, Pontocerebellar Hypoplasia Type 3, Mutation, CRISPR/Cas9

## Abstract

**Background:**

Pathogenic variations in the PCLO gene cause Pontocerebellar Hypoplasia type 3 (PCH3), an extremely rare autosomal recessive disease characterized by seizure, intellectual disability, developmental delay, and microcephaly. PCLO encodes the Piccolo protein, which plays a critical role in synaptic function and neurological disorders. To date, only one pathogenic PCLO variant associated with PCH3 has been reported in the literature. While research on PCH3 is ongoing, the rarity of the condition has limited the number of studies.

**Materials and Methods:**

A novel homozygous variant in PCLO (NM_033026: c.458TC, p. Met153Thr) was identified through wholeexome sequencing and confirmed by Sanger sequencing. Functional studies were conducted to assess the pathogenicity of this variant using next-generation sequencing (NGS), in silico analysis, CRISPR-edited cells, and real-time PCR.

**Results:**

The proband presented with seizure, microcephaly, mild ataxia, and behavioral issues. Notably, in addition to previously reported symptoms, the patient also exhibited toe-walking, loss of tendon reflexes, and unilateral paralysis. The PCLO knockout cell model and molecular analysis confirmed the loss of function of the Piccolo protein in the homozygous variant. Our findings also demonstrated that Piccolo deficiency may affect the expression of other genes, including CtBp1 and BSN.

**Conclusion:**

We identified a novel PCLO variant responsible for PCH3 in a second known family worldwide. Additionally, a CRISPR-based cell model for PCH3 was developed, providing a valuable foundation for further research into the molecular mechanisms underlying Piccolo function and disease pathogenesis.

## Introduction

The PCLO gene is crucial for the proper synaptic function in the brain. Located on chromosome 7q21.11, PCLO encodes the Piccolo protein, which is highly conserved across different species, highlighting its importance in the nervous system [1]. The PCLO gene consists of 25 exons and encodes 5,142 amino acids in Homo sapiens. To date, three isoforms of PCLO have been identified, generated through alternative splicing, all sharing the same start codon [2][3].

Piccolo, Presynaptic cytomatrix protein, is a key component of the cytomatrix at active zones (AZs). Due to its large size and multi-domain structure, Piccolo binds various partners, regulating neurotransmitter release. It also maintains AZ integrity, facilitates F-actin assembly, and aids in synaptic vesicle recycling [4][5]. Piccolo’s function is critical for synapse maintenance and signal transmission in the brain. It interacts with proteins like Bassoon, RIM, Munc13, and ELKS/CAST. Piccolo and Bassoon are common components of both glutamatergic and GABAergic synapses, where they modulate phosphorylation to prevent vesicular protein degradation [6][7].

Variations in PCLO have been linked to various neurological conditions, including epilepsy, schizophrenia, autism spectrum disorders, and Pontocerebellar Hypoplasia type 3 (PCH3) [8][9][10]. PCH3 is a genetic disorder that disrupts the development of the brainstem and cerebellum, leading to developmental delay, progressive microcephaly, brachycephaly, optic atrophy, hypertonia with hyperreflexia, seizures, and short stature. In 2003, PCH3 was reported in a family from Oman, mapped to 7q11-21 [11]. In 2015, the same group identified a pathogenic variant (NM: 033026 c.10624C>T, p.Arg3542Ter) in exon six of PCLO in four siblings from the original pedigree [10]. In this study, we identified a novel PCLO variant as the cause of PCH3 in a second family worldwide and conducted a comprehensive review of the literature. Our detailed characterization revealed previously unreported symptoms.

Given the rarity of PCH3, understanding PCLO and its protein function may offer insights into other neurological disorders. Therefore, the CRISPR/Cas9 system was employed to create a cell model to investigate how PCLO variants affect biological functions, focusing on the expression of two genes related to synaptic function and plasticity. This model provides a strong foundation for future research into Piccolo and related molecular mechanisms.

## Materials and Methods

**Table:** Table1. PCLO Primers for Detection Desired Variant in the Proband and her Parents

c.458T>C, p. Met153Thr	633	AAGAGTTGGATAGTAGTCATC	GTTTAACTGATTCTCCCCTTA

**Table:** Table2. Designed sgRNA Sequences for Human PCLO Gene

Guides	Forward (5’>3’)	Reverse (5’>3’)
Guide1	CACCGAGTGCTCAGGATACCAGGTG	AAACCACCTGGTATCCTGAGCACTC
Guide2	CACCGTACTGTGGAATCTGCCCGG	AAACCCGGGCAGATTCCACAGTAC

**Table:** Table3. PCLO Primers for Evaluation Knockout

PCR product length	Forward (5’>3’)	Reverse (5’>3’)
1051	AACTGCTTCTCCACAAACCACTACA	CATCTTGGCTGTCTTAGGACTTGCT

**Table:** Table4. RT-PCR Primer Sequences

Gene	Forward (5’>3’)	Reverse (5’>3’)
*P* *CLO*	ACCTCAGAGTTTACCTAAAGAAGA	AAGAATATCCCGTGTCGCT
*BSN*	AGAGCCCAGCCAACTATAAC	GCAGTTCAGACAGAGCCA
*CTBP1*	CGGATTGGCAGTGGTTT	AGGTTCAGGATGTGGCA

**Figure-1:**
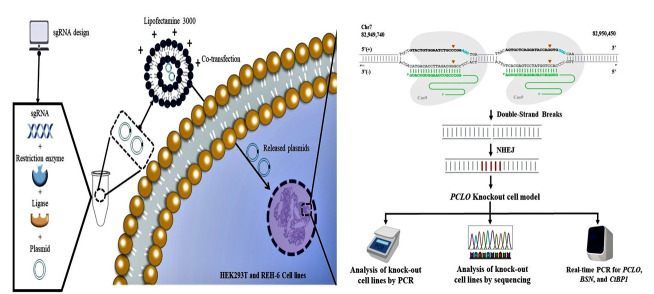
Figure 1. CRISPR/Cas9-Mediated Non-homologous End Joining (NHEJ) for Knockout of the PCLO Gene. Overview of the PCLO gene knockout process in this study, outlining the steps for achieving gene knockout using the CRISPR/Cas9 system .

### 1.Patient Investigation

### 1.1.Ethical Compliance

The subject of the study was a 38-year-old female, born to consanguineous parents, who was evaluated at the Comprehensive Medical Genetics Center in Shiraz, Iran. She, along with her parents, underwent thorough physical examinations. Genetic analysis involved whole exome sequencing (WES) and Sanger sequencing, performed on DNA extracted from their samples. The parents provided written informed consent to participate and publish the findings. The research followed the ethical guidelines established by the World Medical Association's Declaration of Helsinki for studies involving human participants.

### 1.2.Exome Sequencing and Data Analysis

Genomic DNA was extracted from blood samples using a QIAamp DNA Blood Mini Kit (Qiagen, Hilden, Germany) for Whole Exome Sequencing (WES), Sanger sequencing, and additional analyses. The exome sequencing of the proband was conducted using an Illumina NovaSeq6000 (Illumina San Diego, CA), capable of 100-bp paired-end sequencing [12][13].

The raw data were then aligned to the human reference genome (hg19) using the Burrows-Wheeler Aligner [14]. The Genome Analysis Toolkit (GATK) was used to identify single-nucleotide polymorphisms (SNPs). Variants were annotated using the software ANNOVAR [15]. These variants were classified, including pathogenic, likely pathogenic, variant of unknown significance (VUS), likely benign, and benign, following the American College of Medical Genetics and Genomics standards for interpreting sequence variations [16]. Eventually, a homozygous missense variant (NM_033026: c.458T>C, p.Met153Thr) in the PCLO gene was identified in the proband (individual V-1).

### 1.3.Sanger Sequencing and In-silico Analysis

PCR was used to amplify the regions with mutations. Primers were designed specifically for this purpose with the help of Oligo Primer Designer (Table-1) [17]. The DNA amplification involved a series of thermocycling steps, starting with a 15-minute cycle at 95°C, followed by 35 cycles each of 30 seconds at 95°C, 30 seconds at 56°C, and 15 seconds at 72°C, and a final extension at 72°C for 5 minutes. The presence of the variant (NM_033026: c.458T>C, p. Met153Thr) in PCLO was confirmed by analyzing the result of Sanger sequencing using Chromas v2.01.

Various prediction tools, including FATHMM-XF, FATHMM-MKL, EIGEN PC, and SIFT, were employed to evaluate the pathogenicity of the identified variant. FATHMM-XF uses a hidden Markov model for functional annotation. It demonstrated a sensitivity of 89% and a specificity of 81% in benchmarking studies involving missense mutations associated with neurological diseases. EIGEN PC integrates a wide range of annotations to predict the functional significance of variants. It has been reported to achieve a sensitivity of approximately 85% and specificity of 82% for identifying pathogenic missense variants in neurological genes.

Additional in-silico tools such as String and KEGG PATHWAY Database were used to identify related genes and their molecular pathways. Information on the domains of the Piccolo protein was collected from the UniProt database [18].

### 2.Generation of PCLO Knockout Cell Model Using CRISPR/Cas9 Technology

A cell model for Pontocerebellar Hypoplasia Type 3 was designed using the CRISPR/Cas9 system. A schematic of the steps involved in this method is presented in Figure-1.

### 2.1. Designing of Guide RNAs

The target genomic DNA sequences within the exon 2-6 of PCLO in chromosome 7 were analyzed using various CRISPR design tools (http://crispor.tefor.Net, http://chopchop.cbu.uib.no, https://www.synthego.com, http://portals.broadinstitute.org/gppx/crispick//public,

and http://crispr.mit.edu/) to predict two suitable guide RNAs.

These guide RNAs were designed for simultaneous use to expel approximately 652 bp fragments within exon six, resulting in the knockout of the PCLO gene (Table-2).

Then, the designed guide RNA sequences were inserted into specific sites of distinct pSpCas9 (BB)-2A-GFP (PX458) plasmids, employing a standard cloning technique that involves a single-step digestion-ligation. Afterward, these cloned plasmids were transformed into Escherichia coli DH5α competent cells.

The colony PCR was performed to confirm the positive colonies.

These selected clones were subsequently checked through Sanger sequencing [19].

### 2.2.Cell Culture

### 2.2.1.HEK293T Cells

HEK293T cells were cultured in high-glucose DMEM (Gibco) enriched with 10% fetal bovine serum (FBS) (Gibco) and 1% penicillin-streptomycin antibiotics (Sigma-Aldrich) at 37°C with 5% CO2. HEK293T cells were seeded in 6-well plates at a density of 1×106 cells per well and left to adhere for Twenty-four hours resulting in a confluent monolayer.

### 2.2.2.REH-6 Cells

REH-6 cells, derived from a patient with acute lymphocytic leukemia (ALL), were similarly cultured but RPMI 1640 medium (Gibco).

This medium was also enriched with 10% FBS and 1% penicillin-streptomycin. Maintained at 37°C with 5% CO2, the REH-6 cells were prepared for experiments by seeding them in six-well plates at a density of 600,000-800,000 cells per well right before transfection.

### 3. Transfection and FACS Sorting

Lipofectamine 3000 (Thermo Fisher Scientific) was utilized to deliver both cloned constructs into selected cell lines, including HEK293F and REH-6. The cell medium was replaced 16 hours post-lipofection, and cells were further incubated for 48 hours. The efficiency of the transfection was initially estimated under a fluorescence microscope. Then, cells expressing GFP were singled out using a FACSAria III flow cytometer and individually sorted into two 96-well plates for further analysis.

### 4. DNA Extraction, PCR, and Sanger Sequencing

For each cell clone, genomic DNA was extracted using a DNA extraction kit (Favorgen) following the manufacturer’s guidelines. Specific primers, designed using the Oligo Primer Designer, were employed to amplify targeted regions of the DNA and distinguish CRISPR-edited cells from unedited ones. To confirm the deletion, Sanger sequencing was performed on the PCR product obtained from PCLO-homozygous deleted cells. The designed primers are listed in Table-3.

### 5. Real-time PCR Assay

Per the manufacturer's instructions, total RNA from PCLO-knockout and control HEK293T Cells was extracted using the Tissue Total RNA Mini Kit (Favorgen). Then, cDNA was synthesized using First Strand cDNA Synthesis Kit (SinaClon).

The RT PCR was performed on a QuantStudio 3 Real-Time PCR System.

The used primers are listed in Table-4.

All experiments were done in triplicate to ensure accuracy, and the expression levels of the PCLO, BSN, and CTBP1 genes were analyzed using the 2-ΔΔct method [20].

### 6. Ethical Approval

This study is approved by the Ethics committee of Shiraz University of Medical Sciences (IR.SUMS.REC.1400.757).

## Results

**Figure-2:**
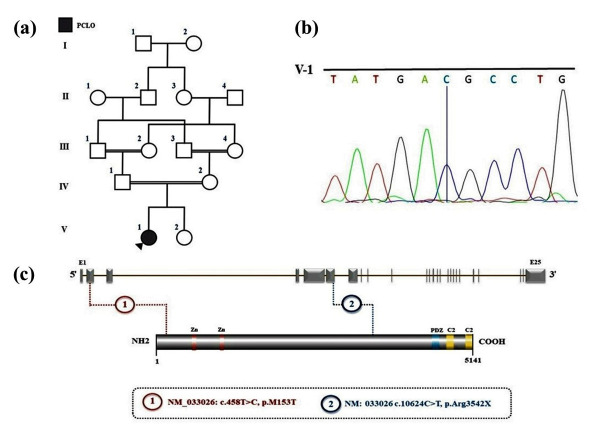
Figure 2. (a) The pedigree of the affected family is shown, with the proband indicated by an arrow. This pedigree

**Figure-3:**
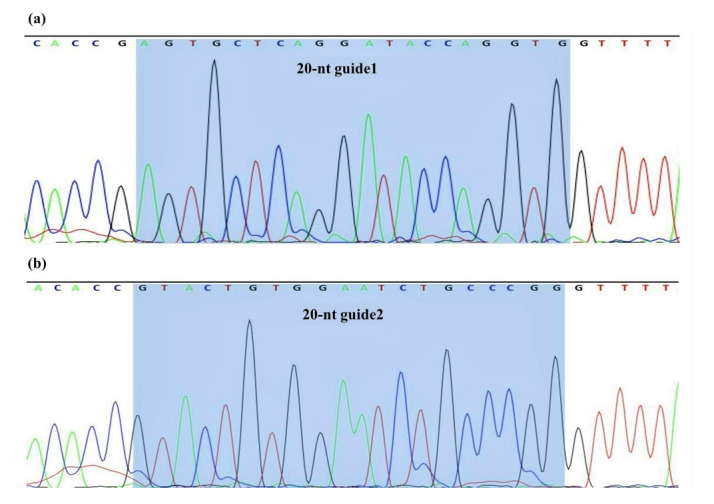
Figure 3. Sanger sequencing results confirming successful cloning of guide RNAs into PX458 vectors using the BbsI restriction enzyme. (a) Electropherogram of guide 1 inserted into the PX458 vector. (b) Electropherogram of Guide 2 inserted into the PX458 vector

**Figure-4:**
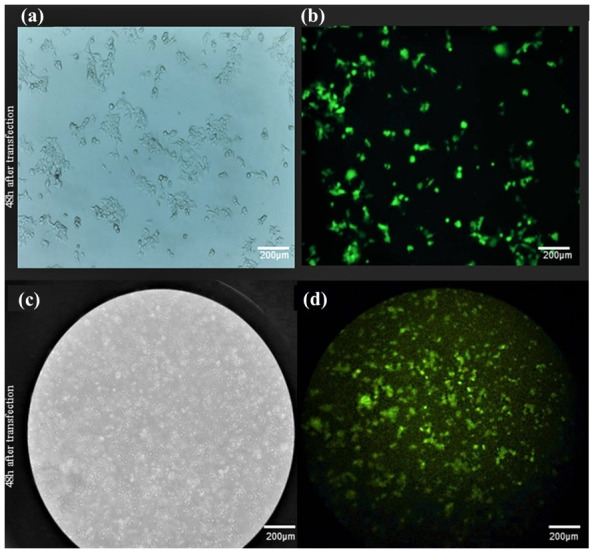
Figure 4. GFP protein expression in HEK293T and REH-6 cells following transfection with Lipofectamine 3000 reagents, demonstrating the incorporation of vectors containing gRNAs in a subset of cells. (a) HEK293T cells under UV light. (b) GFP+ HEK293T cells. (c) REH-6 cells under UV light. (d) GFP+ REH-6 cells.

**Figure-5:**
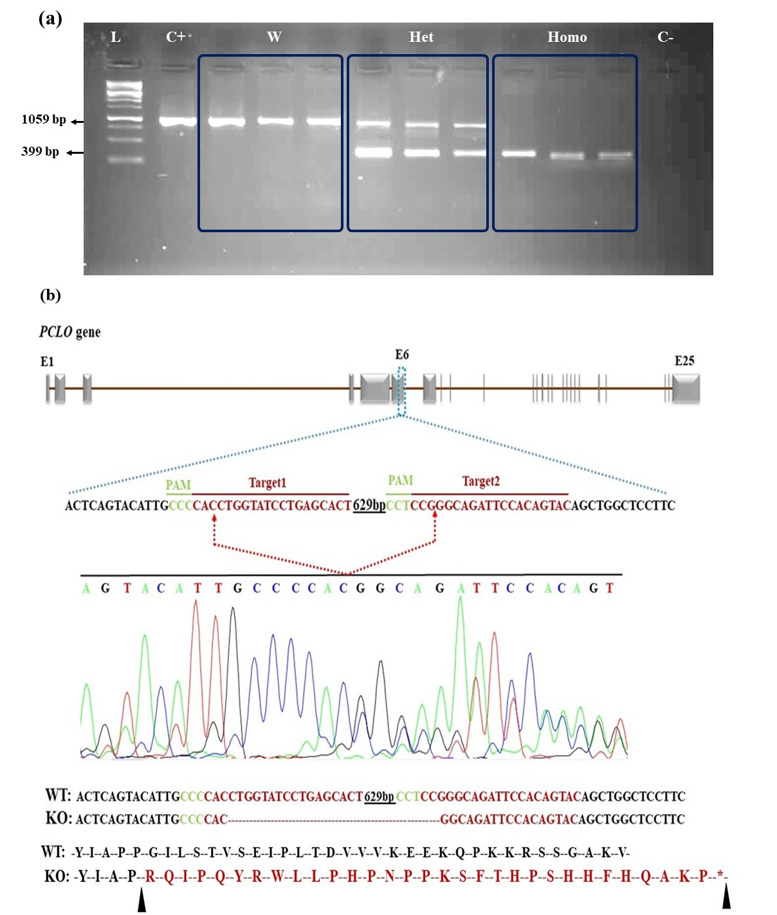
Figure 5. (a) PCR analysis confirms the deletion of an approximately 692 bp segment. Some indel variability among individual clones indicates incomplete Cas9 cleavage efficiency. (b) Sanger sequencing analysis confirms the deletion of an approximately 692 bp segment between the two guide RNAs .

**Figure-6:**
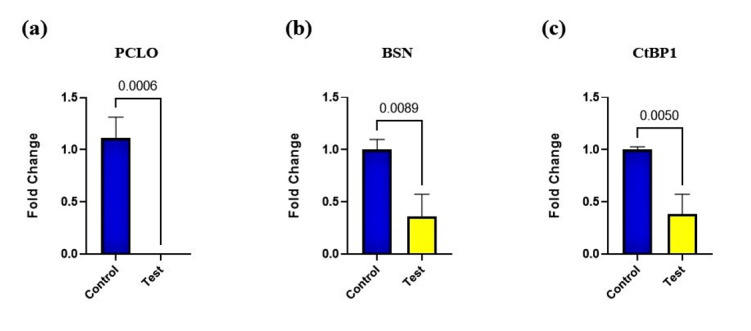
Figure 6. Expression levels of Piccolo, Bassoon, and CTBP1 in Piccolo Knockout (PCLO-KO) and Wild-Type (WT) Cells

**Table:** Table5. Phenotypic Delineation of the Affected Individuals. A Review of the Phenotypic Features of All Previous Cases and Description of Our Patient Reported in This Study

**Cases**	**Case 1**	**Case 2**	**Case 3**	**Case 4**	**Case 5 (our study)**	
**Variant**	c.10624C>T p.Arg3542Ter	c.10624C>T p.Arg3542Ter	c.10624C>T p.Arg3542Ter	c.10624C>T p.Arg3542Ter	c.458T>C p.Met153Thr	
**Inheritance**	AR	AR	AR	AR	AR	
**Gender**	♂	♀	♀	♀	♀	
**Age last assessed**	12 years	6 years	1 year	11 years	38 years	
**Origin**	Oman	Oman	Oman	Oman	Iran	
		**Growth**			
**Prenatal complication**	-	-	-	-	-	
**Mode of delivery**	Natural childbirth	Natural childbirth	Cesarean section	ND	Natural childbirth	
**Last height in cm**	116	90	71	ND	170	
**Last weight in kg**	21	7.4	6	ND	60	
		**Head and neck**			
**Microcephaly**	+	+	+	ND	+	
**Brachycephaly**	+	+	+	ND	-	
**Prominent eyes**	+	+	+	ND	-	
**Low-set ears**	+	+	+	ND	-	
**Gum hypertrophy**	-	+	-	ND	+	
**Uplift of the earlobe**	+	-	-	ND	-	
**Open-mouthed appearance**	-	+	-	ND	-	
		**Neurological features**			
**Seizures**	+	+	+	+	+	
**Seizure onset**	1 year	8 months	6 months	ND	1 year	
**Exaggerated tendon reflexes**	+	+	+	+	-	
**Hypotonia**	Truncal	Truncal	Central	Truncal	-	
**Floppy (since birth)**	+	+	-	+	-	
**Learning disability**	ND	ND	ND	+	+	
**Intellectual disability**	ND	ND	ND	ND	+	
**Loss of tendon reflexes**	-	-	-	-	+	
**Ataxia**	ND	ND	ND	ND	+	
**Speaking ability**	-	+	+	ND	+	
**Optic abnormalities**	Pale optic disk	Optic atrophy, intermittent horizontal nystagmus	ND	ND	-	
**Follow the light**	+	ND	+	+	+	
**Hearing ability**	Could react to loud noises	ND	Failed a hearing test	ND	+	
**Motor ability**	Unable to crawl, sit unsupported, or walk; was able to sit in a wheelchair only for a short period	Spasticity of the limbs; contractures of knees and elbows	Did not show head control or roll over; could grasp objects placed onto her palm; could bring her hand to her mouth	ND	Toe walking; Gait problems; did not develop normal motor abilities	
		**Others features**			
**Frequent respiratory illness **	+	+	+	ND	Frequent sinus infections	
**Cardiac examinations **	Unremarkable	ND	ND	ND	-	
**Muscle bulk**	Appeared Normal	ND	Normal	ND	Normal	
**Other**	Spindle-shaped fingers, markedly irritable, showed dissatisfaction when disturbed	A thin, malnourished child, clubfoot on the left, thoracic scoliosis, an EEG showed sharp discharges from the temporal regions bilaterally, bouts of diarrhea at 8 months, died from an acute respiratory illness at 6 years of age	ND	ND	Paralysis of one side of the body, Autistic spectrum disease	
		**References**			
**Reference**	[10][11]	[10][11]	[10][11]	[10][11]	Present study

### 1. Clinical Features

Our subject (Case 5, Table-5) is a 38-year-old female with mild intellectual disability, microcephaly, muscle weakness, and a history of seizures. She was born via vaginal delivery to healthy consanguineous parents, with no reported perinatal complications or pregnancy difficulties. Her birth weight, head circumference, developmental milestones, and weight gain were all within normal limits. The patient remained asymptomatic and developed normally until the age of 1, when she experienced a febrile seizure. Her parents also reported recurrent sinus infections.

Motor milestones were within normal limits, as she could hold her neck, crawl, sit, roll over, and bear weight without any issues. No delays in sitting or walking were reported. However, the patient now suffers from gait disturbances, including toe walking, and requires assistance for ambulation. Signs of puberty have fully developed.

The patient exhibits feature of ataxia, such as unsteady walking, an inability to walk in a straight line, and difficulty standing with feet together. Although her speech is intact and she passed vision and hearing tests, autistic features have been observed. Neurological examination revealed a loss of tendon reflexes and mild psychomotor impairment. Gum hypertrophy was also noted.

Additional symptoms include intellectual and learning disabilities, right-sided paralysis, and generalized physical weakness. Despite muscle weakness, her muscle bulk remains normal.

### 2. Identification of a Novel PCLO Missense and In-silico Analysis

A homozygous missense variant (NM_033026: c.458T>C, p.M153T) in the PCLO gene was identified in the proband through WES and was verified by Sanger sequencing. Figures-2a and -2b illustrate the family’s pedigree and the electropherogram, respectively. This PCLO variant is associated with PCH3, and the core phenotypes related to PCH3 were obtained from the OMIM database (# 608027).

Bioinformatics tools, including EIGEN PC, FATHMM-MKL, FATHMM-XF, and SIFT, classified this variant as "pathogenic." Notably, this variant is not recorded in the gnomAD browser beta (https://gnomad.broadinstitute.org/) or the ClinVar databases (https://www.ncbi.nlm.nih.gov/clinvar/), indicating its rarity or novel status.

Domains of Piccolo protein were collected from UniProt [21]. The two-dimensional structure of Piccolo was designed using DOG 1.0. [22]. The layout of both previously reported and newly identified variants is illustrated in Figure-2c.

### 3. Generation of PCLO Knockout Cell Model Using CRISPR/Cas9 Technology

### 3.1. Cloning

Following cloning and bacterial culture, E.coli DH5α colonies containing the plasmid constructs appeared on Ampicillin positive LB agar plates due to the Amp resistance marker in the plasmid. Correct insertion of the guide RNAs into PX458 plasmids was verified by Sanger sequencing (Figure-3).

### 3.2. Transfection of Vectors into the HEK293T and REH-6 Cell Lines

To develop a precise cell model of PCH3, transfection was performed on HEK293T and REH-6 cell lines. The transfection efficiency was assessed by counting GFP-expressing cells, revealing an efficiency of approximately 50-60% for the HEK293T cell line and less than 20% for the REH-6 cell line (Figure-4).

### 3.3. Analysis of PCLO Knockout Cell Lines by PCR and Sequencing

PCR was performed on genomic DNA from all expanded clones to determine each PCLO allele's status. Four of the 31 expanded clones exhibited a homozygous deletion in the PCLO gene, and nine exhibited a heterozygous deletion. Further validation of these deletions was achieved through Sanger sequencing of the genetically uniform clones, confirming alterations in exon six of the PCLO gene. Results of the evaluation of CRISPR-mediated PCLO knockout at the genomic level is shown in Figures-5a and -5b.

### 3.4. Real-time PCR

RT-PCR was utilized to assess whether the PCLO gene had been effectively knocked out at the mRNA level in the cells. The results showed a near-complete reduction in PCLO mRNA levels in the homozygous knockout (KO) clones compared to the wild-type controls, as depicted in Figure-6a. Given that Piccolo and Bassoon proteins are highly homologous, the impact of Piccolo knockout on Bassoon was specifically examined. Quantitative real-time PCR (Q-PCR) revealed that mRNA levels of Bassoon were significantly reduced in the Piccolo KO cells, as illustrated in Figure-6b.

The expression of CTBP1, a transcriptional co-repressor, in Piccolo-deficient cells, was also examined using RT-PCR. The findings indicated a significant reduction in CTBP1 levels (Figure-6c). This suggests that the knockout of Piccolo affects not only its immediate homologous proteins but also influential components in broader transcriptional regulatory networks.

## Discussion

To date, only one study has investigated a family with PCH3 and identified a loss-of-function variant in PCLO [10][11]. In this study, we reported a new Iranian patient with PCH3 caused by a novel PCLO variant (NM_033026: c.458T>C, p.Met153Thr). In silico studies and prediction tools such as EIGEN PC, FATHMM-MKL, FATHMM-XF, and SIFT indicate that the variant is likely pathogenic.

A summary of the clinical and genetic findings from all published and newly identified PCH3 patients is provided in Table-5. Common symptoms among the patients include significant seizures, progressive microcephaly, and hypertonia with hyperreflexia. All individuals with pathogenic PCLO variants exhibited seizure (5/5). Hypotonia and exaggerated tendon reflexes were observed in all four previously reported family members (4/5).

Additionally, microcephaly was present in four cases (4/5), and three cases displayed brachycephaly (3/5), though most clinical data of one individual (case 4-table 5) were unavailable. Almost all cases suffered from frequent respiratory illnesses (4/5), with one individual (Case 2, Table-5) dying from an acute respiratory illness at six years of age. Three of the four previously reported cases had prominent eyes, low-set ears, and hypotonia (3/5), whereas our patient exhibited none of these features. Gum hypertrophy was noted in two cases, including our subject (2/5). Hearing loss was reported in only one patient (Case 3, Table-5) (1/5).

Additionally, the first case showed reactions only to loud noises (1/5). Unique to our case were ataxia, loss of tendon reflexes, toe-walking, gait disturbances, unilateral paralysis, and autistic spectrum disorder (1/5).

Most of these clinical features can be attributed to Piccolo’s role as a presynaptic scaffold protein. Piccolo is highly conserved across species and is essential for the regulated assembly and function of synapses [23]. The protein contains a PDZ domain, two zinc finger regions, and two C2 domains [24]. To investigate Piccolo’s function, we generated a full PCLO knockout model using CRISPR/Cas9 technology. The previously reported pathogenic variant resides on exon six of PCLO and is predicted to eliminate PDZ and C2 domains [10].

Based on this, we designed two target sites within exon six of PCLO. The resulting indel mutation in this region introduces a premature stop codon, preventing the production of a functional Piccolo protein.

In 2002, Fujimoto characterized Piccolo in mice and revealed its association with cAMP-GefII and Rim2. The C2A domain of Piccolo is capable of forming homodimers in the presence of Ca2+ and interact with Rim2 or the cAMP GefII-Rim2 complex [25]. Later, Takao-Rikitsu et al. identified that Piccolo, Bassoon, Cast, Rim1, and Munc13-1 form a large molecular complex at the active zone in rat brains [26].

In 2010, Piccolo knock-in/knockout mice were generated. While postnatal mortality increased in Piccolo KO mice, electron microscopy and electrophysiological analysis of Piccolo-deficient synapses did not show a significant phenotype [27]. Recent studies focus on the role of PCLO in PCH3 and its associated phenotype. In 2020, Flack et al. generated Piccolo KO rats, revealing structural changes in the cerebral cortex, cerebellum, and pons, along with behavioral abnormalities such as motor impairments and seizures. Additionally, KO rats showed decreased mossy fiber bouton size, affecting synaptic transmission, confirming that loss of PCLO function may contribute to PCH3-associated phenotypes [28].

Mukherjee et al. explored the roles of Piccolo and Bassoon in neurons by knocking down Bassoon in both wild-type and PCLO KO neurons.

Despite the near-total absence of Piccolo and Bassoon, no electrophysiological changes were detected at glutamatergic or GABAergic synapses. However, electron microscopy showed reduced synaptic vesicle clustering in neurons lacking both proteins. These findings suggest that Piccolo and Bassoon are involved in vesicle clustering but do not directly influence neurotransmitter release [27].

In our study, Bassoon was down-regulated at the mRNA level in the Piccolo KO cell model. Piccolo and Bassoon are components of the CAZ, where neurotransmitter release occurs. Both proteins form Piccolo-Bassoon transport vesicles (PTVs), which serve as CAZ precursor vesicles transported along axons. Piccolo and Bassoon exhibit a high degree of sequence similarity and overlap in their roles in presynaptic function, organizing the CAZ and regulating activity-dependent signaling between presynaptic boutons and the neuronal nucleus.

Piccolo and Bassoon negatively regulate presynaptic autophagy [29]; the absence of both proteins has been shown to increase ubiquitination of presynaptic proteins and reduce the synaptic vesicle pool size [7].

Additionally, studies have shown that Piccolo and Bassoon co-localize in retinal ribbon synapses. Pathogenic variants in BSN have been associated with eye disorders such as night blindness and cone-rod dystrophy (CRD) [30].

Thus, it is possible that the optic atrophy seen in PCH3 patients may be related to decreased Piccolo and Bassoon expression in ribbon synapses.

We also observed a significant reduction in CtBP1 mRNA expression in the Piccolo KO cell model. Previous studies demonstrated altered morphology and expression of ribbon-related proteins in the PCLO-/- mouse model [1].

This led us to hypothesize that CtBP1 expression, a ribbon-associated protein, might also be affected. CtBP1 (C-Terminal Binding Protein 1) is a transcriptional co-repressor found in presynapses and nuclei, playing a role in activity-dependent neuronal gene regulation [28].

It is anchored to the presynaptic cytomatrix through interactions with Bassoon and Piccolo, with neuronal activity regulating its availability for nuclear import. Disorders associated with CTBP1 include hypotonia, ataxia, developmental Delay, Tooth Enamel Defect Syndrome, and chromosomal deletions [31]. Similar symptoms, such as ataxia and hypotonia, have been observed in diseases linked to variations in both PCLO and CTBP1.

Further studies are necessary to understand how PCLO mutations could influence the mechanism underlying PCH3. Our findings suggest that Piccolo deficiency may impact the expression of other genes, including CtBP2, RIM, GIT1, and GIT2. However, more comprehensive studies are needed to confirm these effects, and explore the relationship between these candidate genes and PCLO mutations. This study only proposes these genes as potential candidates for further investigation.

## Conclusion

This study identified a novel PCLO variant associated with Pontocerebellar Hypoplasia Type 3 (PCH3). Additionally, we have utilized CRISPR-Cas9 technology to develop an efficient PCLO knockout cell model. This Piccolo-deficient cell model serves as a valuable tool for studying PCH3, offering theoretical support for the loss of function of the Piccolo protein in cases of homozygous PCLO variants.

Given the complexities of the PCLO gene, further investigations are strongly recommended to deepen our understanding of the pathophysiology, improve diagnostic accuracy, and better characterize the clinical manifestations of PCH3.

## Conflict of Interest

The authors declare that they have no conflicts of interest.
